# MicroRNA Signatures in Cardiometabolic Disorders as a Next-Generation Diagnostic Approach: Current Insight

**DOI:** 10.3390/ijms262110769

**Published:** 2025-11-05

**Authors:** Concetta Iside, Francesca Picone, Paola Di Pietro, Angela Carmelita Abate, Valeria Prete, Antonio Damato, Eleonora Venturini, Saad Akeel, Salvatore Petralia, Carmine Vecchione, Albino Carrizzo

**Affiliations:** 1 Department of Medicine, Surgery and Dentistry “Scuola Medica Salernitana”, University of Salerno, 84081 Baronissi, Italy; ciside@unisa.it (C.I.); aabate@unisa.it (A.C.A.); vprete@unisa.it (V.P.);; 2 Vascular Physiopathology Unit, IRCCS Neuromed, 86077 Pozzilli, Italy; 3 Department of Drug and Health Sciences, University of Catania, Via Santa Sofia 64, 95125 Catania, Italy; saad.akeel@phd.unict.it (S.A.);

**Keywords:** cardiovascular disease, miRNA, diabetes, diagnostic discovery, biomarkers

## Abstract

Cardiometabolic diseases, including cardiovascular disorders and type 2 diabetes mellitus, are the leading cause of morbidity and mortality worldwide, placing a significant burden on healthcare systems. Although advances in imaging and risk stratification have improved disease management, conventional diagnostic and prognostic tools often lack the requisite sensitivity and specificity for early and precise risk stratification. This limitation stems from their poor ability to capture the full molecular complexity of these conditions, underscoring an urgent need for innovative biomarkers to bridge these gaps. MicroRNAs, small non-coding RNAs that regulate gene expression post-transcriptionally, have emerged as promising candidates. Their characteristics offer several advantages over traditional methods, including exceptional stability in biological fluids, strong tissue and disease specificity, and the ability to reflect dynamic pathological changes. These unique features enable miRNAs to detect subtle molecular alterations that may precede clinical symptoms, thereby overcoming key limitations of current diagnostic approaches. Altered circulating miRNA profiles have been linked to pathological processes such as endothelial dysfunction, inflammation, oxidative stress, and maladaptive cardiac remodeling. This review provides a comprehensive overview of the current evidence supporting the diagnostic and prognostic role of circulating miRNAs in cardiometabolic disease. We highlight their potential as early detection biomarkers, tools for patient stratification, and indicators of therapeutic response. Furthermore, we discuss key limitations to clinical translation, including methodological variability, challenges in sample handling, differences in normalization strategies, and platform-dependent quantification inconsistencies. Overcoming these obstacles and achieving robust large-scale clinical validation will be essential to fully harness the potential of miRNAs as next-generation molecular signatures in precision medicine.

## 1. Introduction

The global impact of cardiometabolic disorders extends beyond morbidity and mortality widely, shaping public health priorities and straining healthcare systems [1]. Although substantial progress has been made in refining diagnostic imaging and clinical risk scores, current strategies still fall short in detecting disease at its earliest, subclinical stages. This gap highlights the need for innovative biomarkers capable of capturing the underlying molecular disturbances that precede overt clinical manifestations. While valuable, conventional protein biomarkers such as troponins, B-type natriuretic peptide (BNP), C-reactive protein (CRP), and glycated hemoglobin (HbA1c) often lack the sensitivity and specificity required to detect disease at its earliest stages or to provide information about how the disease progresses and how well the treatment is working [2]. These traditional markers often reflect damage after it has occurred, which highlights the critical need for a new generation of biomarkers that can offer earlier, more precise and mechanistically relevant insights.

Over the last decade, microRNAs (miRNAs) have emerged as a novel class of regulatory molecules with significant diagnostic potential, transforming our understanding of disease pathophysiology [3,4]. These small non-coding RNAs act as master regulators of gene expression at the post-transcriptional level, affecting a wide range of essential biological processes. miRNAs exert their effects through the RNA-induced silencing complex (RISC), which binds to complementary sequences within the 3′ untranslated regions (3′UTRs) of target mRNAs, resulting in translational repression or mRNA degradation [5]. They have a significant impact on cardiometabolic pathophysiology, regulating important processes such as inflammation, fibrosis, angiogenesis, and lipid metabolism. Dysregulation of specific miRNA profiles directly contributes to the development and progression of cardiovascular diseases such as atherosclerosis, myocardial infarction and stroke. The intricate regulatory networks governed by miRNAs have led to the identification of specific miRNA signatures, distinct patterns of up- or down-regulated miRNAs that are uniquely associated with particular conditions. For instance, in atherosclerosis, these signatures can differentiate between stable and unstable plaques, which could enable the prediction of rupture before a severe stroke or heart attack. Studies have revealed that miRNAs such as miR-33 [6], which regulates cholesterol homeostasis, and miR-145 [7,8] which modulates vascular smooth muscle cell proliferation, play a pivotal role in this process. In acute ischemic stroke, changes in circulating miRNAs can be detected within hours of the event, providing a more effective diagnostic tool than traditional imaging [9]. Other studies have shown that altered levels of miRNAs, such as miR-124 [10] and miR-155 [11,12], are associated with neuronal damage and neuroinflammation following an ischemic event. Furthermore, specific miRNAs, such as miR-21, have been shown to be involved in a wide range of pathological processes, including fibrosis and inflammation, making them promising theranostic targets as well as diagnostic markers [13,14].

The complexity arises from the fact that a single miRNA can target hundreds of genes, and a single gene can be regulated by multiple miRNAs, creating an interconnected regulatory network that mirrors the intricate nature of these diseases [15,16].

This review critically examines the current understanding of circulating miRNA signatures in cardiometabolic disorders, highlighting their mechanistic relevance, diagnostic potential, and translational challenges. Furthermore, emerging detection technologies and future directions for clinical integration are discussed.

## 2. Biology of microRNAs

### 2.1. Biogenesis

**Figure 1:**
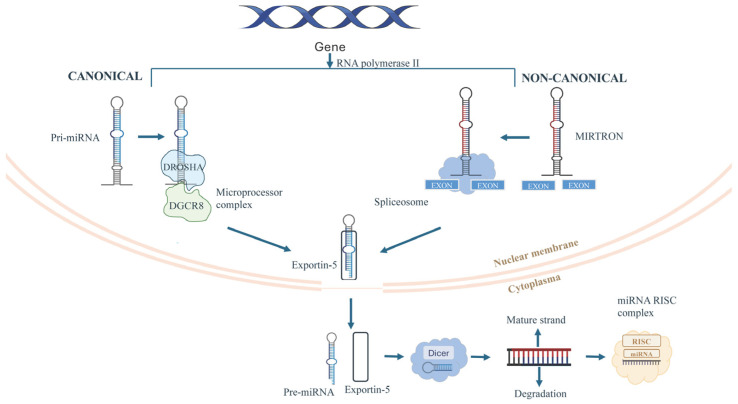
Schematic representation of canonical and non-canonical miRNA biogenesis. Canonical miRNAs are generated from pri-miRNAs processed by the DROSHA–DGCR8 complex, while non-canonical mirtrons arise from spliced introns. Both pathways produce pre-miRNAs that are exported by exportin-5 from nucleus and cleaved by Dicer in cytoplasm, generating mature miRNAs loaded into the RISC complex.

MicroRNAs are small non-coding RNAs of ~22 nucleotides that regulate gene expression post-transcriptionally. They are transcribed by RNA polymerase II as primary transcripts (pri-miRNAs) containing stem-loop hairpins [17]. In the nucleus, Drosha and its cofactor DGCR8 process pri-miRNAs into ~65–70 nucleotide precursor miRNAs (pre-miRNAs) with a 2-nucleotide 3′ overhang [17]. Pre-miRNAs are exported to the cytoplasm, where Dicer, with cofactors such as TRBP or PACT, cleaves the terminal loop to produce a ~21–23 nt miRNA duplex. The guide strand is loaded into Argonaute proteins to form the RNA-induced silencing complex (RISC), which mediates gene silencing via mRNA cleavage or translational repression and decay (Figure 1) [18]. Non-canonical pathways, like mirtrons, bypass Drosha processing through spliced introns that fold into pre-miRNA-like hairpins, highlighting the flexibility and precision of miRNA biogenesis [19]. Dysregulation at any stage of miRNA biogenesis, such as transcriptional control, Drosha/DGCR8 processing, nuclear export, Dicer activity, and RISC loading, has been implicated in pathological processes, including cardiovascular disease, immune dysfunction, and neurodegeneration [17]. For instance, impaired Dicer activity directly inhibits miRNA maturation and is closely linked to cardiac hypertrophy and heart failure. Recent studies demonstrate that disruptive modifications of Dicer, such as through oxidative by-products like 4-hydroxynonenal (4-HNE), suppress its function and lead to reduced miRNA biogenesis, defective cardiac remodeling, and progressive heart failure [20,21]. Furthermore, aberrant Drosha expression or function contributes to endothelial dysfunction and metabolic imbalance. Evidence shows that endothelial-specific deletion of Dicer impairs angiogenesis and promotes heart failure, while altered Drosha/Dicer activity increases expression of the anti-angiogenic factor TSP-1, exacerbating vascular complications observed in diabetes and cardiovascular disease [22,23]. These findings emphasize the pivotal role of precise Dicer and Drosha regulation in maintaining cardiovascular and metabolic health, and underscore their relevance in the pathogenesis of cardiometabolic disorders.

This makes miRNA biogenesis an important key for precision medicine, both in terms of therapeutic intervention and biomarker discovery.

### 2.2. Biological Basis for Diagnostic Utility

MicroRNAs have emerged as clinically relevant biomarkers in cardiometabolic disease, offering distinct advantages over conventional diagnostic tools due to their unique biological characteristics. Their expression profiles exhibit remarkable tissue specificity, which enables the precise identification of the cellular origin of circulating signals. For instance, miR-208a and miR-499 are almost exclusively expressed in cardiomyocytes, serving as highly specific markers of myocardial injury [17,18]. Similarly, miR-126 reflects the status of endothelial cells, miR-122 is a strong indicator of hepatic function, and miR-375 is linked to pancreatic β-cell activity [19,20,21]. This selective enrichment allows for the attribution of organ-specific injury, a level of diagnostic resolution rarely attainable with traditional serum proteins. Furthermore, miRNAs display exceptional temporal sensitivity [22]. They are released into the circulation within hours of cellular stress or injury, often preceding the elevation of conventional biomarkers. In acute myocardial infarction, for example, circulating miR-1 levels can increase as early as two hours after onset, providing crucial diagnostic information before cardiac troponins reach their peak [23]. Finally, the exceptional biochemical stability of circulating miRNAs makes them highly promising as biomarkers. Unlike many other RNA species that degrade rapidly, miRNAs are resistant to RNase activity and harsh conditions like multiple freeze–thaw cycles and pH changes [24]. This protection is afforded by their enclosure within extracellular vesicles such as exosomes and microvesicles or their association with RNA-binding proteins, such as Argonaute-2 [25]. This molecular integrity allows for reliable detection and quantification using established techniques like qRT-PCR. Collectively, this intrinsic durability, along with their tissue specificity and ability to reflect subtle cellular pathology, positions circulating miRNAs as ideal, non-invasive biomarkers for liquid biopsy-based diagnostics (Table 1). Nevertheless, despite their remarkable analytical advantages, several technical and pre-analytical challenges still limit their routine clinical implementation. Variability in sample collection, processing, and storage conditions can markedly influence miRNA yield and integrity. Additionally, hemolysis can introduce confounding signals, particularly for erythrocyte-enriched miRNAs such as miR-451 or miR-16. Moreover, the absence of universally accepted normalization strategies and differences among quantification platforms—such as qRT-PCR, microarray, and next-generation sequencing—further complicate data comparability across studies. Addressing these methodological inconsistencies through standardized protocols and robust reference controls will be crucial to harness the diagnostic potential of circulating miRNAs fully.

## 3. Diagnostic Insights from Cardiovascular miRNAs

Given the complexity of cardiovascular disease, a new generation of diagnostic tools is needed to offer a deeper, more specific understanding of a patient’s condition. miRNAs have emerged as a powerful solution, acting as molecular messengers that not only signal the presence of disease but also reveal its unique signature at the cellular and tissue level. Their expression profiles provide a detailed insight into the heart and vasculature, offering an advanced level of information that is expected to transform the approach to diagnosing, categorizing, and monitoring cardiovascular pathology.

### 3.1. Acute Myocardial Infarction (AMI)

The accurate and timely diagnosis of acute myocardial infarction (AMI) remains a cornerstone of clinical cardiology, where rapid identification directly impacts survival and recovery. While high-sensitivity cardiac troponins (hs-cTn) are the current gold-standard biomarkers, their diagnostic performance can be limited in the very early hours after symptom onset [24]. Circulating miRNAs (c-miRNAs) have therefore emerged as powerful complementary tools, with cardiac-enriched species such as miR-208a, miR-499, miR-1, and miR-133a consistently validated as highly specific indicators of myocardial injury [25]. These miRNAs are rapidly released into the bloodstream, often within 2–4 h of ischemia, preceding detectable rises in hs-cTn, and their levels correlate with infarct size and ventricular dysfunction. Novel candidates such as miR-296-5p, miR-660-3p, miR-107, and miR-101-3p have also demonstrated strong diagnostic power [26]. Their performance is particularly enhanced when combined in multi-marker panels, underscoring the clinical potential of miRNA-based signatures for early “rule-in/rule-out” strategies in patients presenting with chest pain [26]. Beyond these classical markers, new regulatory mechanisms are being uncovered. For instance, the circular RNA circ_0091761 has been shown to be significantly upregulated in AMI patients, promoting endothelial injury by sponging miR-1278, whereas its suppression protects against apoptosis and inflammation, suggesting the circ_0091761/miR-1278 axis as a promising diagnostic and therapeutic target [27]. Similarly, miR-210-3p and miR-582-5p were found downregulated in AMI, with their loss leading to overexpression of MXD1 and the promotion of ferroptosis in hypoxic cardiomyocytes [28]. Their restoration suppressed cell death and provided robust diagnostic accuracy, with combined panels achieving AUCs above 0.80, supporting their role as classifiers of AMI and as predictors of coronary artery disease progression [28]. Elevated levels of miR-142 have been associated with AMI and correlate strongly with cardiac troponin I (r = 0.707), serving as an independent risk factor for the condition.

Additionally, miR-142 predicts worse long-term outcomes and reduced survival in affected patients [29]. Exosomal miRNAs are gaining attention as well, with studies identifying time-dependent increases in panels including miR-3473, miR-504, miR-490-5p, and miR-218a-2-3p in animal and human AMI, enabling machine learning-based diagnostic models with high precision and even accurate estimations of infarction onset time [30]. Among individual biomarkers, miR-1 has been extensively validated through meta-analysis, showing pooled sensitivity and specificity values of 78% and 85%, respectively, and robust diagnostic value across populations and detection platforms [31]. Importantly, miRNAs are not only diagnostic but also therapeutic. Stem cell-derived extracellular vesicles (EVs) and EV-mimetic nanovesicles were shown to confer cardioprotection via delivery of miR-24-3p, which activates the Nrf2 pathway and suppresses cardiomyocyte apoptosis, highlighting translational opportunities for miRNA-based therapies [32]. Finally, novel targets such as miR-409-5p, which is downregulated in AMI and inversely correlated with USP7 expression and cTnI levels, add another promising biomarker to the growing repertoire, with potential applications for both diagnosis and treatment [33].

### 3.2. Coronary Artery Disease and Atherosclerosis

Emerging evidence consistently highlights the diagnostic potential of c-miRNAs and exosomal miRNA profiles as minimally invasive biomarkers across different manifestations of coronary artery disease (CAD) and related cardiovascular conditions such as atherosclerosis [34]. Several studies have shown their ability to capture disease-specific signatures with high accuracy. For instance, exosomal miR-432-5p and miR-382-3p were found upregulated in patients with impaired myocardial perfusion, suggesting their role in linking functional myocardial alterations to molecular signaling pathways [35]. Similarly, the development of advanced biosensors, such as gold metallene-based electrochemiluminescence platforms, has enabled highly sensitive detection of miR-126-3p.

This confirms its downregulation in coronary artery calcification and validates its utility for early screening [36]. Beyond single markers, novel studies employing both conventional qRT-PCR and machine learning approaches have identified miR-140-3p as a robust diagnostic biomarker, able to improve prediction models when integrated with established risk factors [37]. Expanding this concept, multi-miRNA panels, such as a seven-miRNA signature including miR-10b-5p, miR-29c-3p, and miR-486-3p, have demonstrated outstanding diagnostic power in discriminating late-onset CAD (AUC 0.9924), reinforcing the added value of combinatorial biomarker strategies [38]. Population-specific studies further support the role of lipid-related miRNAs. For instance, miR-128-3p is significantly reduced in Indian CAD patients and shows independent predictive capacity even when adjusted for confounders [39], while miR-106b-5p has emerged as a reliable candidate with strong ROC performance (AUC 0.8975) [40]. Beyond canonical miRNAs, isoform microRNA (isomiR) profiling revealed even stronger correlations with coronary calcium burden in NAFLD patients, indicating that sequence variants may surpass conventional miRNAs in diagnostic accuracy [41]. Additional mechanistic insights come from studies linking specific miRNAs to CAD phenotypes: miR-34a, miR-145, and miR-222 can distinguish between obstructive CAD and INOCA, with miR-145 as an independent predictor of non-obstructive forms [42], while EV-derived miR-146b-5p, miR-4701-3p, and miR-1180-3p showed high specificity for subclinical atherosclerosis [43]. Broadly, different works confirm the consistency of findings across contexts, underscoring miRNAs such as miR-19b, miR-186-5p, miR-331, and miR-638 as promising biomarkers for fibrosis, plaque rupture, myocardial infarction, and vascular calcification, particularly when used in multiplex panels [44,45,46]. More recently, specific miRNAs have been tied to acute and prognostic endpoints, such as miR-139-5p in acute coronary syndrome with correlation to troponin and major adverse cardiovascular events [47], miR-210 in the formation of coronary collaterals via VEGF-A/EphrinA3 pathways [48], and exosomal miR-16-2-3p in diabetic coronary microvascular dysfunction through regulation of fatty acid metabolism [49]. In the diabetic context, miR-375 also emerged as a predictor of CAD with fair diagnostic performance (AUC 0.74) [50]. Finally, expanding beyond miRNAs, the interplay between lncRNAs and miRNAs also shows diagnostic promise, as highlighted by the LINC00426/miR-873-5p/SRRM2 axis in atherosclerosis [51]. Taken together, these findings provide a coherent framework in which miRNAs either individually, in panels, or through regulatory networks emerge as powerful, non-invasive biomarkers for CAD and related vascular conditions. They also show strong potential for integration into clinical diagnostics and personalized risk stratification.

### 3.3. Heart Failure

The quest for superior non-invasive diagnostic and prognostic tools in Human Heart Failure (HF) has positioned circulating miRNAs as indispensable molecular components. Evidence from large-scale meta-analyses and human cohort studies highlights the emergence of multi-miRNA panels for accurate patient stratification across the HF spectrum. For instance, a recently validated diagnostic panel discriminates HFpEF patients from both healthy controls and HFrEF with high specificity [52], while another panel comprising eight miRNAs (miR-17-5p, miR-20a-5p, miR-21, miR-23, miR-27, miR-106b-5p, miR-210, and miR-221) has been linked to overall HF incidence, suggesting its utility for early molecular detection prior to clinical onset [53]. In prognostic stratification, the combination of miR-27a-3p, miR-129-5p, miR-145-5p, and miR-590-3p robustly predicts all-cause mortality in HFrEF, yielding a striking hazard ratio (HR) of 4.26 across thousands of participants [53]. Additionally, miR-122-5p and miR-423-5p have been specifically associated with cardiovascular death. Conversely, in HFpEF, circulating miR-19a-3p independently predicts all-cause mortality. Beyond panels, specific miRNAs act as sentinels of key pathological processes. Cardiac remodeling and fibrosis are consistently reflected by elevated miR-21-5p, miR-23a-3p, miR-142-5p, and miR-126-3p [54], with miR-21 and miR-29a levels in hypertrophic cardiomyopathy directly correlating with myocardial fibrosis burden on imaging [55]. Notably, recurrent dysregulation of miR-126, miR-21, miR-145, miR-92a, and miR-155 delineates a vascular injury and inflammation signature in ischemic heart disease [56], underscoring their overlapping roles across cardiovascular phenotypes. Finally, endocrine involvement in HF has been revealed by a panel of miR-10b-5p, miR-193a-3p, and miR-1-3p, which strongly correlates with multiple hormonal deficiencies [57]. Collectively, these validated miRNAs, whether considered as panels or individual biomarkers, demonstrate their multifaceted and recurring involvement in HF pathophysiology, establishing them as essential tools for implementing precision medicine strategies in cardiology.

## 4. Metabolic miRNAs in Diagnosis of Diabetes

Circulating miRNAs are increasingly recognized as pivotal molecular regulators and promising biomarkers across the diabetes–cardiovascular axis, offering unprecedented opportunities for early diagnosis, risk prediction, and therapy personalization. In nutritional intervention research, the CORDIOPREV trial demonstrated that baseline plasma miRNA profiles could stratify patients with newly diagnosed Type 2 Diabetes Mellitus (T2DM) according to their likelihood of remission after five years of dietary intervention: low miR-let7b-3p predicted greater benefit from a low-fat diet, whereas elevated miR-141-5p, miR-182, and miR-192 favored remission under a Mediterranean diet, with composite miRNA–clinical scores enhancing predictive accuracy [58]. Beyond dietary responsiveness, diagnostic applications have been exemplified in T2DM-associated coronary artery disease (CAD), where a three-miRNA panel (hsa-miR-4505, hsa-miR-4743-5p, hsa-miR-4750-3p) achieved outstanding diagnostic performance (AUC = 0.959), surpassing individual miRNAs and offering a robust non-invasive tool [59]. Similar vascular implications were reported in INOCA patients, where upregulation of miR-92a-3p and downregulation of miR-363-5p in diabetic patients provided mechanistic insight into endothelial dysfunction and identified potential therapeutic targets [60].

Extending beyond cardiovascular complications, circulating vesicle-derived miR-378a-3p has been validated in multiple mouse models as a surrogate marker of pancreatic β-cell mass, paving the way for earlier detection of β-cell dysfunction [61]. Similarly, clinical studies identified miR-4454 as significantly reduced in T2DM, correlating inversely with HbA1c, LDL-C, and the presence of nephropathy and hypertension, suggesting both diagnostic and prognostic roles [62]. Consolidating such evidence, a systematic review encompassing 71 studies highlighted 79 dysregulated miRNAs, most notably miR-126 and miR-192, across diabetic complications, reinforcing their consistency as molecular hallmarks and revealing convergence on key pathogenic pathways [63]. Genetic variants within non-coding RNA loci further can modulate disease susceptibility, for example MALAT1 rs619586 exerted protective effects against Type 1 Diabetes Mellitus (T1DM), whereas miR-146a rs57095329 variants increased disease risk and metabolic impairment in pediatric patients [64]. On a broader scale, a large international study integrating 50 miRNAs into a machine learning-derived dynamic risk score (DRS) achieved high predictive power for T1DM progression (AUC = 0.84), with added value in stratifying therapeutic responsiveness, particularly through miR-27b-3p, in clinical trials of imatinib [65]. Earlier CORDIOPREV findings similarly identified a predictive four-miRNA signature (high miR-150 and miR-30a-5p, low miR-15a and miR-375) that anticipated T2DM onset years before diagnosis, providing a practical alternative to burdensome glucose tolerance tests [66]. Finally, pediatric investigations in T1DM revealed 28 dysregulated miRNAs. Most notably, there was upregulation of hsa-miR-101-3p, miR-135a-5p, miR-143-3p, miR-223-3p, and miR-410-3p, and downregulation of miR-495-3p. These miRNAs target VEGFA, IGF-1, and AKT1 pathways, thereby linking altered circulating signatures to vascular and metabolic complications [67].

Together, these findings underscore the multifaceted potential of miRNAs. They function not only as static biomarkers but as dynamic molecular signatures capable of guiding nutrition-based interventions, refining cardiovascular and metabolic diagnostics, anticipating complications, and tailoring therapeutic strategies, thus establishing them as a cornerstone in the evolution of precision medicine for diabetes and its cardiovascular sequelae (Table 2).

## 5. Inflammatory Crosstalk and Shared miRNA Signatures in Cardiometabolic Diseases

### 5.1. Anti-Inflammatory Regulatory miRNAs

One of the hallmarks of cardiometabolic diseases is the persistent low-grade inflammation that underlies the bidirectional link between metabolic dysregulation and vascular injury. The persistent inflammatory milieu is accountable for the pathogenesis of insulin resistance, endothelial dysfunction, and atherosclerosis, in addition to orchestrating organ remodeling processes such as cardiac fibrosis. Within this network, circulating miRNAs are critical mediators and putative biomarkers, which reflect the balance between protective and pathogenic immune networks [68,69]. Among the migratory miRNAs, a subpopulation confers potent anti-inflammatory effects, modulating immune function, endothelial function, and tissues remodeling. Of these, miR-146a, miR-223, and miR-126 have been consistently proven to modulate chronic inflammation in cardiometabolic disease as key regulators in metabolic and vascular homeostasis. These miRNAs not only reflect the underlying inflammatory state but also hold promise as diagnostic biomarkers and therapeutic targets for warding off diabetes pathogenesis, atherosclerosis, and cardiac remodeling [70]. MiRNA-146a is a significant anti-inflammatory negative feedback process targeting predominantly the NF-κB pathway through downregulation of TRAF6 and IRAK1 and therefore suppressing innate inflammatory responses [71]. In atherosclerosis, miR-146a suppresses macrophage apoptosis and generation of proinflammatory cytokines in response to the oxidized LDL and TLR activation. Aside from its anti-inflammatory effect, miR-146a also has direct cardioprotection. Intravenous administration of a miR-146a mimic after an acute myocardial infarction improves cardiac performance, reduces scar volume, and preserves viable myocardial tissue These effects are mediated, in part, through the regulation of NADPH Oxidase 4 (NOX4) and the induction of endothelial nitric oxide synthase (eNOS) expression [72,73]. Circulating miR-146a levels also correlate with metabolic health and oxidative balance, increasing in physically active individuals and decreasing in sedentary or metabolically impaired subjects [74,75]. MiRNA-223 is another essential anti-inflammatory miRNA that regulates cardiovascular inflammation by influencing T-helper cell differentiation and macrophage polarization and thus orchestrates the progression of atherosclerosis and diabetic cardiomyopathy [76]. In NSTE-ACS patients, higher miR-223 is associated with reduced myocardial injury and improved prognosis, proving to be a diagnostic marker for plaque stability [77]. In functional manners, miR-223 regulates endothelial inflammation, macrophage phenotype, and suppresses NLRP3 inflammasome activation [78], while exosomal miR-223 participates in immune modification and tissues repair [79]. In murine models, miR-223 represses inflammation and fibrosis, promotes angiogenesis, and curbs procoagulant activities, suggestive of protective actions in cardiometabolic contexts [80]. Clinically, miR-223 is always reduced in T2DM plasma and microvesicles, consistent with disease progression from prediabetes to diabetes [81]. Talking about vasculoprotective and anti-inflammatory miRNAs, one example par excellence is miR-126. Widely expressed in endothelial cells, miR-126 modulates endothelial growth, angiogenesis, and growth factor signaling to maintain vascular homeostasis. In T2D, the circulating concentrations of miR-126 are significantly reduced, correlating with endothelial dysfunctions and macrovascular complications risks elevation [82]. Downregulation is also observed in atherosclerosis and CAD, and thus miR-126 is a potential early vascular impairment biomarker [83]. Mechanistically, miR-126 modulates adhesion molecules such as VCAM-1, inhibiting leukocyte recruitment and reducing chronic inflammation [83].

### 5.2. Pro-Inflammatory and Remodeling-Associated miRNAs

On the other hand, several miRNAs have been shown to play a pivotal role in inflammation progression and tissue remodeling, thereby contributing to the exacerbation of cardiometabolic complications. Among these, miR-155, miR-21, miR-34a, and miR-132 are of particular significance as key pathogenic mediators, integrating immune dysregulation with structural and functional alterations across the cardiometabolic system [70]. Together, these shared miRNA signatures underline the close link between inflammation, metabolism, and organs damages, positioning them not only as mechanistic mediators but also as promising next-generation diagnostic tools for patients’ stratification in cardiometabolic disorders. A growing body of evidence highlights miR-155 as a central pro-inflammatory miRNA in cardiometabolic diseases, particularly in the context of atherosclerosis and metabolic syndrome (MetS). In atherosclerosis, the accumulation of lipids within the vascular intima is closely associated with local inflammation, where macrophages play a pivotal role in regulating lipid homeostasis and immune activation [84]. MiR-155 has been identified as a critical regulator of inflammatory responses driven by macrophages, functioning through multiple signaling pathways that promote plaque formation, apoptosis, and dyslipidemia [85]. As demonstrated by experimental models, the expression of miRNA-155 has been shown to be significantly increased in the aortas of Western died-field mice, thus contributing to the development of larger atherosclerotic lesions, oxidative stress, and chronic inflammation [86]. Mechanistically, the action of miR-155 is to suppress protective molecules such as Bmal1 and HBP1, thereby promoting ROS production and amplifying inflammatory processes [87]. Beyond atherosclerosis, dysregulated miR-155 expression has been documented in metabolic disorders. Clinical and experimental studies consistently report reduced circulating and tissue levels of miR-155 in patients with MetS, type 2 diabetes (T2D), obesity, and coronary artery disease (CAD), correlating with insulin resistance, hyperglycemia, dyslipidemia, and systemic inflammation [88]. Furthermore, miRNA-21 has been identified as a central regulator of inflammation and fibrosis in cardiometabolic disorders, particularly in cases of diabetic cardiomyopathy (DCM) and age-related cardiac dysfunction [89]. In diabetes models, mice lacking miR-21 exhibit reduced cardiac hypertrophy and enhanced cardiac function in comparison with their wild-type diabetics controls. Conversely, the over-expression of miR-21 has been observed to increase fibrosis and reduce autophagy via the miR-21/SPRY1/ERK/mTOR pathway [90]. Furthermore, elevated levels of circulating miR-21 have been observed in elderly patients diagnosed with cardiovascular disease, and these levels have been shown to correlate positively with inflammatory markers such as C-reactive protein and fibrinogen [89]. Mechanistically, miR-21 drives fibrosis through two majors pathways, the miR-21/TGF-β1/Smad7 axis, where miR-21 promotes TGF-β1 synthesis while suppressing Smad7, thus sustaining fibroblast activation and ECM deposition [91]; and the miR-21/Spry1 pathway, where downregulation of Spry1 by miR-21 enhances ERK–MAP kinase signaling, leading to fibroblast survival, TGF-β1 secretion, and interstitial fibrosis [92].

In addition to miR-155 and miR-21, other microRNAs play significant roles in pathological remodeling. MiRNA-34a is strongly induced by myocardial stress and contributes to ventricular remodeling after myocardial infarction (MI) by inducing cardiomyocyte apoptosis, pathological hypertrophy, cellular senescence and extracellular matrix dysregulation [93]. miR-34a-5p suppresses the cardioprotective role of preconditioning by suppressing syntaxin 1A in hypoxia/reoxygenation models [93]. Similarly, miR-132 is upregulated in stressed cardiomyocytes, promoting pathological remodeling by impairing autophagy, enhancing hypertrophic signaling and disrupting calcium handling [94]. Its targets include the anti-hypertrophic transcription factor FOXO3 and calcium-cycling genes such as SERCA2A, which results in contractile dysfunction and progressive heart failure. Crucially, inhibiting miR-132 in preclinical models prevents adverse remodeling and improves cardiac function, highlighting its potential as a therapeutic target [95]. Combined, miR-155, miR-21, miR-34a and miR-132 emerge as central pro-inflammatory and pro-remodeling mediators in cardiometabolic disease (Table 3). By integrating immune dysregulation, fibrotic remodeling, apoptosis and impaired metabolic signaling, they establish a pathogenic link between chronic inflammation and multi-organ dysfunction.

## 6. Clinical Translation and Diagnostic Platforms of Circulating miRNAs

The clinical translation of circulating microRNAs (miRNAs) as cardiovascular biomarkers is still at an early stage, mainly due to both technical and validation hurdles. From a diagnostic standpoint, several analytical platforms are available, each with distinct advantages and limitations. Real-time quantitative PCR (qRT-PCR) is still considered the gold standard for miRNA detection, offering high sensitivity and specificity, but its limited multiplexing capacity hampers its application to broad biomarker panels [97]. Microarrays allow the simultaneous profiling of hundreds of miRNAs and are widely used in discovery studies. However, their semi-quantitative performance and poor reproducibility across platforms remain critical drawbacks. Next-Generation Sequencing (NGS) represents the most comprehensive and unbiased tool, enabling the discovery of novel and low-abundance miRNAs, but its cost, analytical complexity, and bioinformatic demands prevent routine use in clinical laboratories [98]. Digital PCR (dPCR) has recently emerged as a promising alternative, providing absolute quantification and improved reproducibility compared with qRT-PCR; however, the lack of standardized protocols and limited diffusion in hospital laboratories still restricts its translational potential [99].

Equally important are pre-analytical variables, such as sample type (plasma vs. serum), hemolysis, RNA extraction, and normalization strategies, that strongly influence results and contribute to inconsistencies across studies [100]. Even in conditions where miRNA panels have shown diagnostic potential, such as acute myocardial infarction or chronic heart failure, their incremental value over established biomarkers and clinical scores remains to be convincingly demonstrated [101]. Machine learning approaches, by integrating complex miRNA datasets, offer the possibility of developing more accurate diagnostic and prognostic models, but their robustness depends on large, externally validated cohorts, which are still scarce [102]. Looking forward, incremental progress is expected from two directions: technical innovation aimed at simplifying and standardizing detection platforms, and clinical studies designed as multicenter, prospective validations. In the near future, the integration of dPCR and targeted NGS into routine workflows, combined with bioinformatic tools optimized for reproducibility, could accelerate the path from bench to bedside. While circulating miRNAs represent one of the most promising classes of liquid biopsy biomarkers, their full clinical adoption will ultimately depend on demonstrating clear superiority, or complementary value, to current diagnostic standards.

## 7. Conclusions

Circulating microRNAs represent a new frontier in the diagnosis and management of cardiometabolic diseases. Thanks to their high tissue specificity, rapid response to pathological stress and remarkable stability in body fluids, they have the potential to complement or even surpass conventional biomarkers. There is an increasing body of evidence that highlights their ability to detect early subclinical alterations and to provide valuable information on disease phenotypes, progression and therapeutic response in conditions such as atherosclerosis, acute myocardial infarction, heart failure and diabetes. Their dual nature, acting both as molecular effectors of disease pathways and as measurable diagnostic indicators, places them at the intersection of mechanistic biology and clinical practice, offering an opportunity for precision medicine. By capturing dynamic processes such as endothelial dysfunction, vascular smooth muscle cell remodeling, metabolic imbalance, and inflammatory activation, miRNAs provide a multidimensional picture that extends beyond traditional biomarkers. Particularly relevant is the immuno-inflammatory dimension, where miRNAs orchestrate the crosstalk between innate and adaptive immune responses, amplifying or resolving vascular injury and metabolic stress. This adds an additional layer of diagnostic and prognostic value, as immune-related miRNA signatures may distinguish subclinical from overt disease and even predict therapeutic responsiveness.

Across cardiometabolic and vascular disorders, the evidence supporting specific miRNA signatures varies. Among many candidates, miR-126 and miR-21 show the most consistent and reproducible links with endothelial health, oxidative stress regulation, and cardiovascular outcomes across different groups and analyses. miR-210 also has strong prognostic value in conditions related to hypoxia, such as myocardial ischemia and stroke. In contrast, miR-155 and miR-34a display variable regulation, often depending on the inflammatory stage or tissue-specific context. Conflicting results are especially noticeable for miR-146a, whose regulation differs between acute and chronic vascular injuries. The differences in analytical methods, normalization techniques, and sample types (serum vs. plasma vs. tissue) further complicate comparisons across studies. Overall, endothelial-derived miRNAs (miR-126, miR-21, miR-210) stand out as the most reliable and clinically relevant biomarkers, while inflammatory miRNAs (miR-155, miR-34a, miR-132) tend to be more context-dependent, functioning better as indicators of disease activity rather than long-term prognosis. However, despite these promising findings, several limitations continue to hinder their routine application in clinical settings. Current challenges include significant heterogeneity in pre-analytical variables such as sample collection, storage, and RNA isolation; variability in detection methods across laboratories; and a lack of universally accepted normalization strategies.

Furthermore, many studies are still limited by small sample sizes, single-center designs and population-specific biases, which restrict the generalizability of findings. Another critical issue is the lack of large-scale, prospective validation studies that can demonstrate actual clinical utility in real-world practice as well as statistical significance.

Future research should focus on overcoming these barriers by standardizing analytical pipelines internationally, carrying out rigorous multicenter validation and incorporating cutting-edge technologies such as next-generation sequencing, digital PCR and isomiR profiling. In parallel, integrating multi-miRNA panels with advanced computational approaches, including machine learning and artificial intelligence, could enhance predictive accuracy further and enable the development of robust diagnostic and prognostic algorithms. Furthermore, translational efforts should explore the therapeutic potential of miRNAs by investigating whether modulating their expression could benefit patients with cardiometabolic disorders.

Overall, while the field is still in a transitional phase between the laboratory and clinical practice, circulating microRNAs have the potential to transform the diagnostic landscape of cardiometabolic diseases. By enabling earlier detection, more refined risk stratification, and personalized therapeutic monitoring, these molecular signatures could ultimately help reduce the global burden of cardiovascular and metabolic diseases on patients and healthcare systems.

One of the most provocative questions is whether circulating miRNAs, given their early and dynamic expression changes, could one day be monitored in real time outside the laboratory, perhaps even in home-based settings through miniaturized biosensors or wearable technologies. Such an advance would shift miRNAs from being static diagnostic markers to becoming continuous indicators of gene expression dynamics, enabling pre-symptomatic detection, personalized risk forecasting, and adaptive treatment adjustment. While this scenario remains speculative, it underscores the transformative potential of miRNAs as true “sentinels” of cardiometabolic health and raises the challenge of bridging molecular biology with next-generation digital health solutions.

## Figures and Tables

**Table 1:** Key Properties of Circulating miRNAs as Cardiometabolic Biomarkers.

Key Property	Description (Mechanism & Key Features)	Clinical Utility & Impact
Stability	RNase-resistant; protected by Extracellular Vesicles (Exosomes), Microvesicles, or association with proteins like Argonaute-2	Enables reliable, non-invasive “liquid biopsy” (plasma/serum) with high tolerance for handling variability (e.g., freeze–thaw cycles)
Tissue Specificity	Expression profiles are highly unique to the cell/organ of origin., e.g., Myocardial-specific (miR-208a, miR-499), Endothelial (miR-126)	Allows precise localization of injury or disease state (e.g., distinguishing cardiac stress from hepatic dysfunction)
Temporal Kinetics	Rapid release into circulation (within hours) following acute stress or injury (both active secretion and passive release)	Provides potential for ultra-early diagnosis in acute events (e.g., AMI, preceding Troponin peak) and tracking disease progression over time
Mechanistic Relevance	Core post-transcriptional regulators of key processes: inflammation, fibrosis, lipid metabolism, endothelial function, cardiac remodeling	Offers functional insights into disease pathogenesis, facilitating patient stratification and the identification of therapeutic targets
Networking Capability	One miRNA regulates multiple genes; easily analyzed in Multi-miRNA Panels (Signatures) for a comprehensive view	Significantly boosts diagnostic accuracy and prognostic power compared to single-protein markers, aiding in complex risk stratification

**Table 2:** Schematic summary of miRNA and their diagnostic performance in cardiometabolic disorders.

Disease	miRNA(s)	Regulation	Diagnostic Accuracy (AUC)	Reference
**AMI**	miR-210-3p + miR-582-5p (combined panel)	↓ Downregulated	AUC > 0.80	[28]
**AMI**	miR-1	↑ Upregulated	Sensitivity 78%, Specificity 85% (meta-analysis)	[31]
**CAD**	7-miRNA panel (miR-10b-5p, miR-29c-3p, miR-486-3p + others)	Mixed	AUC = 0.9924	[38]
**CAD**	miR-106b-5p	↓ Downregulated	AUC = 0.8975	[40]
**T2DM-associated CAD**	hsa-miR-4505, hsa-miR-4743-5p, hsa-miR-4750-3p (panel)	↑ Upregulated	AUC = 0.959	[59]
**Diabetes (T2DM)**	miR-375	↑ Upregulated	AUC = 0.74	[50]
**Type 1 Diabetes (T1DM)**	Machine learning panel (50 miRNAs, key miR-27b-3p)	Mixed	AUC = 0.84	[65]

**Table 3:** Representative miRNAs in Cardiometabolic Disorders.

miRNA	Molecular Mechanisms	Biological Effects	Clinical Implications	References
miR-146a	↓ TRAF6, ↓ IRAK1 → NF-κB inhibition; ↓ NOX4; ↑ eNOS	↓ Pro-inflammatory cytokines; ↓ macrophage apoptosis; ↑ endothelial function; ↓ oxidative stress; cardioprotection	Protection against atherosclerosis; improved post-MI recovery; better glycemic control; biomarker of metabolic health	[71,72,73,74,75]
miR-223	Modulates T-helper cell differentiation; macrophage polarization (M1 → M2); ↓ NLRP3 inflammasome;	↓ Inflammation; ↓ fibrosis; ↑ angiogenesis; ↓ procoagulant activity; plaque stabilization	Protective in atherosclerosis and diabetic cardiomyopathy; reduced myocardial injury in NSTE-ACS; biomarker of plaque stability; ↓ levels in T2DM progression	[77,78,79,80,81]
miR-126	Regulates endothelial growth and angiogenesis; modulates VCAM-1 and adhesion molecules; growth factor signaling	Maintains vascular homeostasis; ↓ leukocyte recruitment; ↓ chronic inflammation	Reduced levels in T2D, CAD, hypertension; biomarker of early vascular impairment; therapeutic target in endothelial dysfunction	[82,83]
miR-155	Targets Bmal1; HBP1 → ↑ ROS; activates NF-κB, ERK, STAT3; regulates macrophage inflammatory signaling	↑ Inflammation; ↑ apoptosis; ↑ lipid accumulation; ↑ oxidative stress	Promotes atherosclerosis and MetS; correlates with insulin resistance and dyslipidemia; biomarker of plaque progression	[84,85,86,87,88]
miR-21	miR-21/SPRY1/ERK/mTOR pathway; miR-21/TGF-β1/Smad7 axis	↑ Fibroblast activation; ↑ ECM deposition; ↓ autophagy; ↑ hypertrophy	Drives cardiac fibrosis and remodeling in DCM and aging; circulating marker in CVD; therapeutic target for anti-fibrotic strategies	[96]
				[90,91,92]
miR-34a	Targets syntaxin 1A, SIRT1; activates senescence and apoptosis pathways	↑ Cardiomyocyte apoptosis; ↑ pathological hypertrophy; ↑ ECM dysregulation; ↑ cellular senescence	Promotes ventricular remodeling post-MI; biomarker of stress-induced cardiac injury	[93]
miR-132	Targets FOXO3, SERCA2A; impairs calcium handling; ↑ hypertrophic signaling	↑ Hypertrophy; ↓ autophagy; ↑ contractile dysfunction; adverse remodeling	Upregulated in heart failure; inhibition improves cardiac function; therapeutic candidate against pathological remodeling	[94,95]

## Data Availability

No new data were created or analyzed in this study. Data sharing is not applicable to this article.
